# 55059 Developing the COmmuNity kNowlEdge to aCtion Toolkit (CONNECT) to reduce breast cancer screening disparities Let’s CONNECT

**DOI:** 10.1017/cts.2021.591

**Published:** 2021-03-30

**Authors:** David Haynes, Kelly Hughes

**Affiliations:** 1 University of Minnesota Institute for Health Informatics; 2 Minnesota Department of Health

## Abstract

ABSTRACT IMPACT: Advanced spatial analysis techniques are used to target a community education intervention for Immigrant and African American women to increase breast cancer screening. OBJECTIVES/GOALS: We are addressing breast cancer screening disparities through the development of the COmmuNity kNowlEdge to aCtion Toolkit (CONNECT). CONNECT implements a mixed-methods approach using GIScience, community education, and social media to mitigate the impact of breast cancer screening disparities for Immigrant African and African American women. METHODS/STUDY POPULATION: We used advanced spatial analysis techniques, Spatially Adaptive Filters (SAF) to reveal mammography screening rates below the state level. SAF create screening maps of This new information allows lay health educators to identify and engage their communities in the Breast Cancer Champions program. We transformed and curated existing cancer educational material into culturally relevant educational training for lay health educators. Lay health educators participate in educational trainings and receive stipends for conducting formal and informal breast cancer education and screening events. RESULTS/ANTICIPATED RESULTS: We have identified four principles for designing culturally relevant education materials.

1. Visual representation of the community in materials
2. Positive Framing
3. Statistics and graphs should be minimal
4. Appropriate reading level and minimizing jargon

Due to COVID-19, our breast cancer champions are engaging with their community in socially responsible ways (i.e., engaging through social media, developing and placing community education flyers, community radio spots). Our social media campaign, which began in October has already attracted over 1000 followers. DISCUSSION/SIGNIFICANCE OF FINDINGS: Despite the disruption of COVID-19, our project continues reduce breast cancer screening disparities. We have developed and created culturally appropriate materials and are currently training Champions. By incorporating an online presence into our community outreach, we are increasing the ways we connect with our community.

